# Respiratory viral infections drive different lung cytokine profiles in pigs

**DOI:** 10.1186/s12917-020-02722-8

**Published:** 2021-01-06

**Authors:** Hanna Turlewicz-Podbielska, Ewelina Czyżewska-Dors, Małgorzata Pomorska-Mól

**Affiliations:** 1 Department of Preclinical Sciences and Infectious Diseases, Faculty of Veterinary Medicine and Animal Science, Poznan University of Life Sciences, Wołyńska 35, 60-637 Poznań, Poland; 2 Department of Swine Diseases, National Veterinary Research Institute, Partyzantów 57, 24-100 Puławy, Poland

**Keywords:** Swine influenza A virus, Porcine reproductive and respiratory syndrome virus, Co-infection, Local immunity, Cytokine

## Abstract

**Background:**

Swine influenza A virus (IAV) and porcine reproductive and respiratory syndrome virus (PRRSV) are considered key viral pathogens involved in the porcine respiratory disease complex. Concerning the effect of one virus on another with respect to local immune response is still very limited. Determination of presence and quantity of cytokines in the lung tissue and its relation to the lung pathology can lead to a better understanding of the host inflammatory response and its influence on the lung pathology during single or multi-virus infection. The aim of the present study was to explore and compare the patterns of lung cytokine protein response in pigs after single or dual infection with swine IAV and/or PRRSV.

**Results:**

Inoculation with IAV alone causes an increase in lung concentration of IFN-α, IFN-ɣ, TNF-α, IL-6, IL-8 and IL-10, especially at 2 and 4 DPI. In PRRSV group, beyond early IFN-α, IFN-ɣ, IL-6, IL-8 and IL-10 induction, elevated levels of cytokines at 10 and 21 DPI have been found. In IAV+PRRSV inoculated pigs the lung concentrations of all cytokines were higher than in control pigs.

**Conclusions:**

Current results indicate that experimental infection of pigs with IAV or PRRSV alone and co-infection with both pathogens induce different kinetics of local cytokine response. Due to strong positive correlation between local TNF-α and IL-10 concentration and lung pathology, we hypothesize that these cytokines are involved in the induction of lung lesions during investigates infection. Nevertheless, no apparent increase in lung cytokine response was seen in pigs co-inoculated simultaneously with both pathogens compared to single inoculated groups. It may also explain no significant effect of co-infection on the lung pathology and pathogen load, compared to single infections. Strong correlation between local concentration of TNF-α, IFN-ɣ, IL-8 and SwH1N1 load in the lung, as well as TNF-α, IL-8 and PRRSV lung titres suggested that local replication of both viruses also influenced the local cytokine response during infection.

## Background

Porcine respiratory disease complex (PRDC) is a serious health problem in pork production worldwide [1, 2]. This syndrome can be produced by various combinations of viral and/or bacterial agents. However, swine influenza A virus (IAV) and porcine reproductive and respiratory syndrome virus (PRRSV) are considered crucial viral pathogens involved in the PRDC [3–5]. Porcine reproductive and respiratory syndrome virus is an etiological agent of the porcine reproductive and respiratory syndrome (PRRS) [6]. Some of the hallmarks of PRRSV infection in pigs, which are crucial in the pathogenesis of PRRS, are suppression of type I interferon production and modulation of host immune response [7]. Swine IAV is an etiological agent of swine influenza (SI). The disease is characterised by low mortality (1–2%) and high morbidity (up to 100%) [8]. The increase in production of many proinflammatory cytokines is distinctive for acute influenza in humans, and is known as “cytokine storm” [9–11] This phenomenon is considered extremely important in the pathogenesis of SI [12–14].

Some reports regarding concurrent infection of pigs with various respiratory viruses, including swine IAV and PRRSV were published previously [15–18]. So far, experimental studies dealing with PRRSV and IAV single or dual infections conducted in conventional pigs have been focused on clinical manifestation and production performance, however with numerous clinical outcomes with respect to dual infection [16–18].

Regardless of the results of previous co-infection studies, our understanding of the effect of one virus on another with respect to local immune response is still very limited. Proinflammatory cytokines are believed to play an important role in respiratory infections in pigs by coordinating and activating the adaptive immune response [19, 20]. However, if cytokine level is too excessive, tissue damage and exacerbation of the disease outcome can occur [21]. Therefore, determination of presence and quantity of cytokines in the lung tissue and its relation to the lung pathology can lead to a better understanding of the host inflammatory response during single or multi-virus infection in pigs.

To date, only one *in vitro* study investigated the impact of concomitant PRRSV and SIV infections on various targeting various genes (pathogen recognition receptors, interferons type I, cytokines, and IFN-inducible genes) and proteins [22]. Mentioned study showed an impact of PRRSV/IAV co-infection and superinfections on the cellular and tissue immune response at the molecular level [22]. Other *in vivo* study, on the local innate immune response in bronchoalveolar lavage fluid (BALF) cells of pigs singly inoculated with PRRSV or co-inoculated with PRRSV and IAV revealed that infection with PRRSV alone or with IAV affected the expression of IFN-α and delayed the onset of IFN-γ expression. In addition, co-infection with both viruses demonstrated additive effects on the mRNA expression of IL-6 and IL-10 [23]. Gene expression is often interpreted in terms of protein levels but results of various experiments revealed that the correlations between expression and secretion are not very strong [24, 25]. It seems that mRNA levels are often not reflected in protein levels [24–26].

Therefore, the aim of the present study was to explore and compare the patterns of lung cytokine protein response in pigs after single or dual infection with swine IAV and/or PRRSV. For this, lung samples (supernatants) collected at various time of infection from pigs were analysed with the use of species-specific ELISA assays in order to acquire a better understanding of porcine respiratory viral single and dual infections.

## Methods

### Animals and infection with PRRSV and/or IAV strains

The lung samples used in the study were collected during an animal challenge experiment described elsewhere [27].

Pigs were inoculated intranasally with IAV (avian-like H1N1, isolated from the pig suffering from acute swine influenza (A/Poland/Swine/14,131/2014; SwH1N1) and/or PRRSV (strain PL15-33, subtype 1 PRRSV, isolated from a lung obtained from a pig with respiratory clinical signs from a Polish herd). Virus propagation was carried out as previously described [27].

In short, fifty six 7-week-old healthy conventional, free of influenza A and PRRS viruses and seronegative against these pathogens piglets, not vaccinated against SI and PRRS were randomly divided into four experimental groups (PRRSV, IAV + PRRSV, IAV, CONTROL (14 pigs in each group). Pigs were also seronegative for Aujeszky’s disease virus and *Mycoplasma hyopneumoniae*. No evidence of streptococcosis or atrophic rhinitis was found based on clinical, serological and pathological examinations. Animals were purchased from a local breeding farm (Borysów, Poland). The experimental schedule is presented in Table 1.

**Table 1:** Experimental scheme

Group	Inoculation (IN)	Number of pigs sacrificed at the following time point (DPI)				
	Day 0	2	4	10	21	Total (from 2 to 21)
Control	PBS	3	3	3	5	14
IAV	SwH1N1 10^7^TCID_50_	3	3	3	5	14
PRRSV	PRRSV 10^5^TCID_50_	3	3	3	5	14
IAV + PRRSV	SwH1N1 10^7^TCID_50_ PRRSV 10^5^TCID_50_	3	3	3	5	14

*IN *intranasal, *DPI *day post inoculation, *IAV *pigs inoculated with swine influenza A virus, *PRRSV *pigs inoculated with porcine reproduction and respiratory syndrome virus, *IAV + PRRSV *pigs co-inoculated with PRRSV and IAV

All pigs were housed at the BSL3 animal facility in four independent units. Feed and water were offered *ad libitum*.

On day 0, piglets from IAV and IAV + PRRSV groups were inoculated intranasally (IN) with SwH1N1 (10^7^TCID_50_) in 2 ml of phosphate-buffered saline (PBS). Piglets from PRRSV and IAV + PRRSV groups were inoculated IN with PRRSV (10^5^TCID_50_) in 2 ml of PBS. Mock-inoculated pigs (with PBS) served as controls. Individual pigs were subjected to daily clinical examination and measurement of rectal body temperature (from day 7 pre inoculation until day 21 post inoculation (DPI) or until euthanasia (at 2, 4 and 10 DPI). The severity of clinical lesions was assessed based on a scoring system adapted to PRRS and SI [27]. A clinical scoring system with predefined humane endpoints was used to prevent undue suffering. An overall, well-being, respiration, eye disorders and normal rectal temperature and appetite were scored as 0 (normal condition) to 1 (the most severe disorders in each category). The scores for individual pigs in each category were added up to a cumulative clinical score (CS) per animal. During the experiment no mortality was recorded and none of the animals displayed the acute clinical signs defined as endpoint criteria. Euthanasia of the pigs was performed on 2, 4, 10 and 21 DPI by intravenous injection of pentobarbiturate (50 mg/kg) followed by exsanguination by cutting arteria axillaris. Complete necropsy was carried out on each animal, with special emphasis on the respiratory tract.

Gross lung lesions were scored according to method described by Halbur et al. [28] and adapted by Pomorska-Mól et al. [27]. The observed lesions were described in details in Pomorska-Mól et al. [27]. In summary, each lung lobe was assigned a number to reflect the approximate volume percentage of the entire lung represented by that lobe, to reach the total of 100 possible points. The evaluation according to the above mentioned procedure gave a lung score (LS) to estimate the percentage of the lung affected by pneumonia.

### Local cytokines concentration

Lung tissue collected from control and infected pigs during necropsy was prepared in PBS (pH 7.4) [8, 27]. 1.0 g of lung tissue collected from cranial, middle and accessory right lobes (3.0 g in total) was suspended in 3 ml of PBS (1:1 w/v) and frozen before being homogenized. Next, the samples were centrifuged at 12,000 rpm for 10 min. The supernatants were collected and stored at − 80ºC up to maximum 1 month. The cytokines concentrations were analyzed with the use of porcine cytokine ELISA kits. Porcine IL-8, IL-10, IFN-α and TNF-α from Invitrogen Corporation (Camarillo, USA); Porcine IL-1β and IFN-ɣ from RayBiotech, Inc. (Norcross, USA); IL-6- Pig ELISA Kit from Abcam (Cambridge, UK). The detection limits of kits are: 6 pg/ml (IL-1β), 45 pg/ml (IL-6), 10 pg/ml (IL-8), 3 pg/ml (IL-10), 2 pg/ml (IFN-ɣ), 2 pg/ml (IFN-α) and 3 pg/ml (TNF-α). All tests were run according to the manufacturers’ recommendations. Calculation of the quantity of the cytokines was based on standard curve for each cytokine with the use of FindGraph software.

### Lung pathogen load

Virus titration (SwH1N1 and PRRSV) of lung homogenates was carried out as described elsewhere [27]. The detection limit was equal 1.7 TCID50. PRRSV RNA was isolated from lung tissue homogenate according to method described previously [27]. The results were expressed as copy number/g of tissue. The analytical sensitivity of reaction reached 4 copies of viral RNA per reaction. The reaction was linear within a 101–105 copies/reaction range.

### Statistical analysis

The obtained data were subjected to the W. Shapiro-Wilk test for normality and the Levene’s test for equality of variances. Differences between means were tested for statistical significance by a nonparametric Kruskal-Wallis test with post hoc multiple comparisons for comparison of all pairs. For analysis of correlation between lung cytokine concentration and lung gross lesions (expressed as lung score) and pathogen load the Spearman-Rank correlation were used. Differences were considered as significant with α < 0.05. All calculations were performed with Statistica 13.3 (Tibco, USA).

## Results

### Local cytokine response

**Fig. 1:**
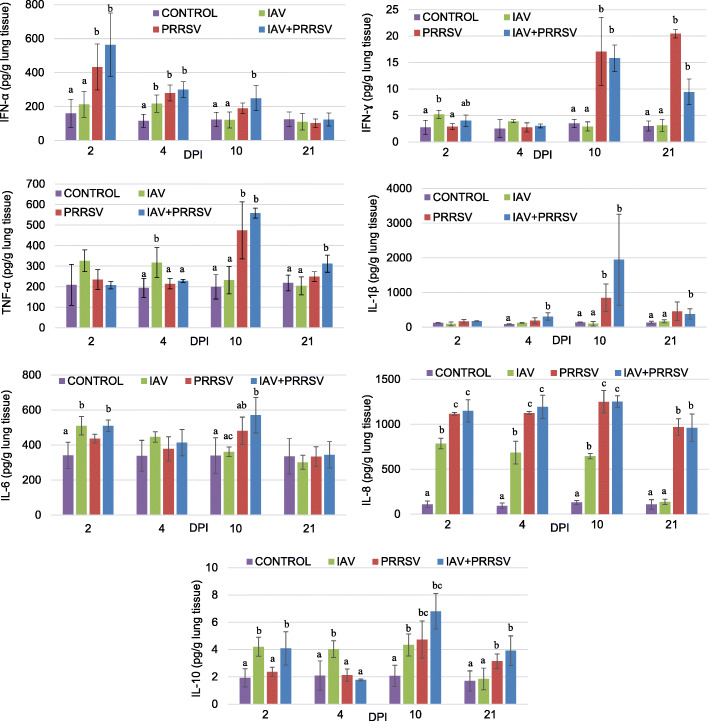
Quantification of cytokines in lungs of control pigs (CONTROL) and pigs inoculated with IAV or PRRSV or co-inoculated with both viruses (IAV + PRRSV) (mean ± SD) at 2, 4, 10 and 21 DPI **a, b, c** - different superscript indicate significant difference between groups at *p* < 0.05

The detailed results regarding the mean (± SD) cytokines concentrations in the lung tissue of experimental pigs are summarised in Fig. 1.

In general, the lung concentrations of investigated cytokines were induced in inoculated and co-inoculated pigs. In PRRSV-inoculated pigs the significantly higher level of IFN-α, IFN-ɣ, IL-1β, IL-10, TNF-α and IL-8 were noted at various time after inoculation, while in only SIV-inoculated pigs the concentrations of IFN-α, IFN-ɣ, TNF-α, IL-6, IL-8 and IL-10 in the lung were elevated (*p* < 0.05) mostly at early stages after inoculation, from 2 to 4 dpi. In co-inoculated pigs elevated level of all investigated cytokines were found compared to control animals (*p* < 0.05).

#### Interferons

The concentration of IFN-α was elevated mainly at the early stage of experimental infections (at 2 and 4 DPI). The highest levels of this cytokine in the lungs were observed at 2 DPI in pigs inoculated with PRRSV or co-inoculated with IAV and PRRSV. At this time-point, the mean concentration of IFN-α was significantly higher in both mentioned groups compared to controls and pigs inoculated only with SIV (*p* < 0.05). At 4 DPI the concentration of IFN-α was significantly higher in all inoculated groups comparing to control group, and no differences were found between inoculated groups (*p* ≥ 0.05). In co-inoculated animals the significant increase of IFN-α in the lung was observed also at 10 DPI.

In IAV-inoculated pigs the levels of IFN-ɣ did not change during the study with exception of day 2 post inoculation (*p* < 0.05). Starting from 10 DPI the significantly higher concentration of IFN-ɣ was noted only in pigs inoculated or co-inoculated with PRRSV. The mean concentrations of this cytokine were particularly high at 10 DPI in PRRSV and IAV + PRRSV. At 4 DPI the mean level of IFN-ɣ were similar to the level observed in control pigs (*p* ≥ 0.05).

#### Interleukins

In the case of IL-1β significantly higher concentration was observed mainly in pigs co-inoculated (at 4, 10 and 21 DPI), while in PRRSV-single inoculated pigs only at 10 DPI.

The mean concentration of IL-6 was elevated in IAV inoculated and co-inoculated pigs at 2 DPI (*p* < 0.05). In contrast at 10 DPI elevated concentrations of IL-6 were demonstrated in PRRSV- and co-inoculated animals (*p* < 0.05). At 4 and 21 DPI no significant differences were found compared to control group (*p* ≥ 0.05).

At 2 DPI, the level of IL-10 was higher only in IAV or PRRSV + IAV inoculated animals (*p* < 0.05) as compared to control group. Significant differences were also observed at 4 DPI between IAV and controls (*p* < 0.05). At 10 DPI the level of this cytokine was the highest in the lung of co-inoculated pigs, but in all inoculated groups the level of IL-10 was still significantly higher than in control animals. At 21 DPI the mean concentrations of IL-10 were significantly higher only in IAV + PRRSV group compared to control animals (*p* < 0.05).

#### The TNF superfamily

The significant changes regarding TNF-α were observed at 4 DPI (IAV), 10 DPI (PRRSV and IAV + PRRSV) and 21 DPI (IAV + PRRSV). In SIV-inoculated pigs slightly elevated levels of this protein were observed also at 2 DPI however without statistical significance comparing to control group.

#### Chemokines

In all inoculated animals the significantly higher concentration of IL-8, compared to controls, was observed from 2 to 10 DPI, while at 21 DPI this increase was significant only in PRRSV inoculated/co-inoculated animals (*p* < 0.05). Furthermore, at 2, 4 and 10 DPI the lung level of IL-8 was significantly higher in group inoculated/co-inoculated with PRRSV comparing to singly-IAV inoculated group (*p* < 0.05).

The correlation between lung cytokine concentration and lung scores is presented in Table 2. Strong, significant correlation was found between mean concentration of TNF-α and IL-10 in the lung and LS. Significant correlations between aforementioned parameters were also confirmed for IFN-ɣ, IL-1β and IL-8 and changes in the lungs.

**Table 2:** The relationship between concentration of cytokines in the lung parenchyma and gross lung lesions (LS) after single or dual inoculation with SIV and/or PRRSV

Lung score	Spearman's rank correlation coefficient (R-Spearman)						
	IFN-α	IFN-ɣ	TNF-α	IL-1β	IL-6	IL-8	IL-10
	0.01	0.51*	0.69*	0.42*	0.17	0.37*	0.60*

^*^a statistically significant correlation with a *p*-value less than 0.05

The correlation between lung pathogen load and cytokine concentration is presented in Table 3. Strong, significant correlation was found between mean lung concentration of TNF-α, IFN-ɣ and IL-8 and SwH1N1 titre in the lung. Significant correlations were also confirmed for TNF-α and IL-8 and PRRSV lung titre, respectively for co-inoculated and single inoculated group.

**Table 3:** The relationship between concentration of cytokines in the lung parenchyma and lung pathogen load after single or dual inoculation with IAV and/or PRRSV

Group	Pathogen load	Spearman’s rank correlation coefficient (R-Spearman)						
		IFN-α	IFN-ɣ	TNF-α	IL-1β	IL-6	IL-8	IL-10
IAV	SwH1N1	0.43	0.77*	0.86*	0.08	0.45	0.62*	0.40
PRRSV	PRRSV	0.07	0.02	0.33	0.42	0.35	0.62*	0.33
IAV + PRRSV	PRRSV SwH1N1	0.41 0.23	0.37 0.81*	0.54* 0.77*	0.09 0.59	0.04 0.23	0.38* 0.63*	0.30 0.20

*IAV *pigs inoculated with swine influenza A virus, *PRRSV *pigs inoculated with porcine reproduction and respiratory syndrome virus, *IAV + PRRSV *pigs co-inoculated with PRRSV and IAV; * a statistically significant correlation with a *p*-value less than 0.05

### Lung lesions and pathogen load

Lung lesions characteristic as well as pathogens load for each group of pigs have been described in details elsewhere [27].

## Discussion

This study reveals additional data generated from an animal experiment conducted as part of the Polish National Science Centre (DEC-2014/13/B/NZ6/02566) project related to studies on the acute phase response and pathogenesis of selected viral, bacterial and mixed infections of the respiratory tract in pigs [27]. In the present report, the effect of single or dual inoculation with IAV and PRRSV on the lung cytokine response has been described. The aim of a present study was to shed a light on the local cytokine response during the course of infection with PRRSV or/and IAV strains of actually present in Poland as well as Central and Western Europe. The biological material analysed in the present study was collected in the animal experiment with the use of such strains [27].

Both pathogens are known to play a crucial role in PRDC [5]. There are several papers confirming the role of SIV and PRRSV as a potent inducer of inflammatory mediators in pig respiratory system [14, 18, 23, 29, 30]. However, to the best of authors’ knowledge, this is the first attempt to investigate and compare the effect of single infection with IAV and PRRSV and co-infection with both pathogens on cytokine secretion profile in the lung and its correlation with lung lesions and pathogen load.

The data published previously revealed that respiratory signs due to IAV infection are not only the result of direct tissue damage by replicating virus but are also related to the proinflammatory local cytokine response [14, 31, 32]. Respiratory signs during infection with PRRSV are also the result of pathology in the lung and interaction with the host immune system, including induction of proinflammatory cytokines secretion [21]. In addition, PRRSV predisposes pigs to co-infection by other respiratory viruses, due to destruction of pulmonary tissues or cells [6, 33].

As mentioned earlier, cytokines play an important role in the immunopathology of porcine viral respiratory infections [14, 18, 21, 23, 30, 34, 35]. When host is infected by various viruses the secretion of cytokines is up- or downregulated [31]. The concentrations of cytokines in BALF have been correlated with viral replication and clinical signs as well as exacerbated lung lesions and neutrophil infiltration [18, 31]. Usually the degree of pathological lesions is not positively correlated with the number of virus particles in the infected tissues, but it may be the result of immunologic injury mediated by the immune response, including cytokine secretion [21, 36]. In the present study concentrations of investigated cytokines were elevated in the lungs of the single or dual inoculated pigs compared to the controls. The highest concentrations of cytokines were observed at various time-point depending on virus used for inoculation. We found the positive, significant correlation between mean concentration of INF-ɣ, TNF-α, IL-1β, IL-8 and IL-10 in the lung and macroscopic lesions (LS) observed in this organ. Previous reports investigating immunopathology of IAV infection have shown a positive correlation between elevated level of IL-1β and neutrophil recruitment to the lungs which may lead to the more severe inflammation [35, 37]. The high concentration of IL-8 may also contribute to the more severe lesions [14]. According to Damjanovic et al. [38] TNF-α is critically required for negatively regulating the extent of lung immunopathology during acute influenza infection. The correlation between lung level of this cytokine and lung pathology may confirm its involvement in the pathomechanism of lung lesions development during viral infections. The significantly higher concentrations of IL-6 in the lungs of IAV inoculated pigs were observed at early stages of infections (at 2 DPI). In contrast, in pigs inoculated or co-inoculated with PRRSV the elevated level of IL-6 was observed also at 10 DPI. Lauder et al. [39] reported that IL-6 has a crucial role and ability to limit inflammation, promote protective adaptive immunity and prevent fatal immunopathology. The early secretion of this cytokine may therefore constitute an important line of defence against a fatal course of SI and possibly other viral respiratory infections. The role of IL-10 during respiratory infection, especially acute influenza appears to be contradictory. Sun et al. [40] found that inhibition of IL-10 secretion during influenza resulted in increased inflammation and decreased survival, whereas McKinstry et al. [41] reported that inhibition of IL-10 signalling before infection enhanced viral clearance and increased survival. The strong positive correlation between mean IL-10 lung level and LS observed in the present study seems to confirm the observations of Sun et al. [40]. The concentration of IL-10 in pigs inoculated/co-inoculated with PRRSV was significantly elevated at 10 and 21 DPI, while in co-inoculated animals at 2, 10 and 21 DPI. In agreement with our study, several previous studies had shown that PRRSV infection, particularly at active stage, resulted in systemic and local production of the immunosuppressive cytokine IL-10 [42, 43].

Interferon-α can be produced in response to virus infection, and viruses have a larger ability to induce production of this cytokine than bacteria [44]. In our experiment the concentration of IFN-α in pigs inoculated with PRRSV or co-inoculated with IAV + PRRSV was elevated earlier than in IAV only inoculated pigs. Liu et al. [21] in the study which examined two different PRRSV isolates, suggest that virus isolate which induced the IFN-α response at later point, depressed the inflammatory reaction. According to the above mentioned findings, the early appearance of IFN-α may exacerbate the inflammatory reaction, which may be one of the reasons for the difference in the lung cytokine response in our study. In groups inoculated with PRRSV with an earlier IFN-α response, a stronger inflammatory response was observed in the later phase of infection.

A previous study evaluating immune and inflammatory response in pigs during acute SI demonstrated significant increase in IL-1β, IL-6, IL-8, IL-10, TNF-α and IFN-γ following IAV infection [14]. The main difference between the present and aforementioned study regards the concentration of IL-1β. In the above-mentioned experiment IL-1β levels were significantly elevated during the entire study (from 2 to 10 DPI). The difference in cytokine profiles may be due to different isolate as well as route of inoculation (intratracheal) and the type of specimen used for analysis (trachea and lung tissue vs. only lung tissue).

Overall, the profile of changes in lung cytokine levels in the present study reflects the pathogenesis of both infections. The presence of lung lesions typical for IAV infection was observed earlier, mostly up to 10 DPI, while lesions typical for PRRSV infection were found later, from 10 DPI. In the only-IAV inoculated group, the highest levels of cytokines were also observed at early stages of infection (2–4 DPI), which corresponded well to the most severe pathological lung lesions. Similarly, in the group inoculated solely with PRRSV, the most pronounced cytokine changes were also associated with the period when severity of lung lesions was the highest (from 10 to 21 DPI).

It should be emphasized that there was no synergistic effect between IAV and PRRSV in terms of effect on local cytokine response. This may be associated with different pathogenesis of both infections, especially in the context of the time of the onset of lesions in the lungs. In the case of SI, lung lesions appear earlier and disappear sooner, while in the case of PRRS the intensity of lung lesions is highest during the period when influenza-related lesions disappear.

In conclusion, current results indicate that experimental infection of pigs with IAV or PRRSV alone and co-infection with both pathogens were able to induce local lung inflammatory response. However, different kinetics of local cytokine response was observed in pigs inoculated with IAV or PRRSV. Because strong positive correlation between local TNF-α and IL-10 concentration and lung pathology has been observed, we hypothesize that these cytokines are involved in the induction of lung lesions during PRRS and SI. Nevertheless, no apparent increase in lung cytokine response was seen in pigs co-inoculated simultaneously with both pathogens compared to single inoculated groups. It may also explain no significant effect of co-infection on the lung pathology and pathogen load, compared to single infections. Strong correlation between local concentration of TNF-α, IFN-ɣ, IL-8 and SwH1N1 load in the lung, as well as TNF-α, IL-8 and PRRSV lung titres suggested that local replication of both viruses also influenced the local cytokine response during infection.

## Data Availability

The datasets used during the current study are available from the corresponding author on reasonable request.

## Abbreviations

BALF: Bronchoalveolar lavage fluid
CS: Clinical score
DPI: Days post inoculation
H1N1: Hemagglutinin type 1 and Neuraminidase type 1
IAV: Swine influenza A virus
IFN-ɣ: Interferon-gamma
IFN-α: Interferon-alpha
IL-10: Interleukin-10
IL-1β: Interleukin-1 beta
IL-6: Interleukin-6
IL-8: Interleukin-8
LS: Lung score
PBS: Phosphate-buffered saline
PRDC: Porcine respiratory disease complex
PRRSV: Reproductive and respiratory syndrome virus
SD: Standard deviation
SI: Swine influenza
SwH1N1: Swine-origin H1N1 influenza virus
TCID: Median tissue culture infectious dose
TNF-α: Tumor necrosis factor alpha
